# Analysis of Polycyclic Aromatic Hydrocarbon in Airborne Particulate Matter Samples by Gas Chromatography in Combination with Tandem Mass Spectrometry (GC-MS/MS)

**DOI:** 10.1155/2021/6641326

**Published:** 2021-05-26

**Authors:** Nam Vu-Duc, Lan Anh Phung Thi, Thuy Le-Minh, Lan-Anh Nguyen, Huong Nguyen-Thi, Loan-Ha Pham-Thi, Van-Anh Doan-Thi, Huong Le-Quang, Hung Nguyen-Xuan, Thao Thi Nguyen, Phuong Thanh Nguyen, Dinh Binh Chu

**Affiliations:** ^1^Center for Research and Technology Transfer, Vietnam Academic of Science and Technology, 18 Hoang Quoc Viet, Hanoi 100000, Vietnam; ^2^School of Environmental Science and Technology, Hanoi University of Science and Technology, No. 1 Dai Co Viet, Hanoi 100000, Vietnam; ^3^FPT University, Hoa Lac High Tech Park, Km 29 Thang Long Boulevard, Thach That, Hanoi 100000, Vietnam; ^4^Department of Analytical Chemistry, School of Chemical Engineering, Hanoi University of Science and Technology, No. 1 Dai Co Viet Road, Hanoi 100000, Vietnam

## Abstract

Polycyclic aromatic hydrocarbons (PAHs), the family of organic contaminations, have been shown to have negative effects on human health. However, until now, the comprehension on occurrence, distribution, and risk assessment of human exposure to PAHs has been limited in Vietnam. In this work, a capillary gas chromatography coupled with electron impact ionization tandem mass spectrometry (GC-EI-MS/MS) has been introduced for analysis of 16 PAHs in some particulate matter samples. PAHs have been separated on the TG 5 ms capillary gas chromatographic column and detected by tandem mass spectrometry in multiple reaction monitoring mode. The PAHs in the particulate matter (PM 2.5 and PM 10) samples were extracted by ultrasonic-assisted liquid extraction and cleaned up by an acidic silica gel solid phase extraction. The linearity range of all analyzed PAHs was from 5 to 2000 ng mL^−1^ with *R*^2^ ≥0.9990. Limit of detection (LOD) of PAHs in particulate matter sample was from 0.001 ng m^−3^ (Br-Naph) to 0.276 ng m^−3^ (Fln). The recovery of PAHs was investigated by international proficiency testing samples. The recoveries of PAHs in proficiency testing sample ranged from 79.3% (Chr) to 109.8% (IcdP). The in-house validated GC-EI-MS/MS method was then applied to analysis of some particulate matter samples that were collected in the Hanoi areas. The total concentrations of PAHs in several brands of samples collected from Hanoi were found in the range of 226.3 ng m^−3^–706.43 ng m^−3^. Among the studied compounds, naphthalene was found at high frequency and ranged from 106.5 ng m^−3^ to 631.1 ng m^−3^. The main distribution of the PAHs in particulate matter samples was two-ring and three-ring compounds.

## 1. Introduction

Polycyclic aromatic hydrocarbons (PAHs) are a family of organic pollutants containing two or more fused aromatic rings [1–4]. The major (90%) PAHs are byproducts of incomplete combustion [5] and man-made activities such as traffic activities, cooking, and fossil fuel burning [6]. Therefore, PAHs can easily leach out into the environment over time and also migrate to human body through three pathways such as air inhalation, dermal contact, and dietary intake. The deleterious health effects of PAH exposure to human body have been reported in several previous studies in the world. In particular, benzo[a]pyrene (BaP) is a first compound which can lead to be cancer [7–9]. Due to the serious impact of PAHs on human health and ecosystem, the United States of America-Environmental Protection Agency (US-EPA) has introduced 16 PAHs including naphthalene (Naph), acenaphthylene (Acy), acenaphthene (Ace), fluorene (Fln), phenanthrene (Phe), anthracene (Ant), pyrene (Pyr), benzo[a]anthracene (BaA), chrysene (Chr), benzo[b]fluoranthene (BbF), fluoranthene (Fluo), benzo[k]fluoranthene (BkF), benzo[a]pyrene (BaP), indeno [1,2,3-cd]pyrene (IcdP), dibenzo[a,h]anthracene (DahA), and benzo[g,h,i]perylene (BghiP) on the list of priority organic pollutants to be removed [4, 10, 11]. Besides, seven PAHs (BaP, BaA, BbF, BkF, Chr, IcdP, DahA) were given in the list of mutagenic and carcinogenic compounds by IARC [12]. In Vietnam, PAHs have been found in widespread matrices, including environment samples (such as air, dust, soil, sediment) and food samples (smoked meat, tea, etc.). These studies have shown that the PAHs pollution has been associated with urbanization and industrialization, especially fossil fuel-based transportation activities. In addition, PAHs are nonpolar compounds (chemical structures of PAHs are shown in Figure S1, Supplementary Information) and could then be bioaccumulated in the animals and then enter the human body through a food chain.

Recently, many studies have shown use of gas or liquid chromatography in combination with mass spectrometry such as gas chromatography in combination with electron impact ionization mass spectrometry (GC-EI-MS) [9, 10, 13], gas chromatography in combination with electron impact ionization tandem mass spectrometry (GC-EI-MS/MS) [14–16], liquid chromatography-atmospheric pressure photon ionization tandem mass spectrometry (LC-APPI-MS/MS) [17–19], liquid chromatography-fluorescent detector in serial with tandem mass spectrometry (LC-FLD-MS/MS) [20] for analysis of PAHs and their derivative compounds such as nitro-PAHs and hydroxyl-PAHs in both environmental and food sample matrices. Among these methods, GC-EI-MS/MS with excellent selectivity, high sensitivity, and robustness has been a modern and effective method for PAHs analysis in several laboratories. In addition, several sample preparation techniques such as ultrasonic-assisted liquid extraction [2, 11, 15], traditional Soxhlet extraction, solid phase extraction (SPE) [16, 21], pressured solvent extraction (or accelerated solvent extraction, ASE) [10, 13], and QuEChERS [9, 16, 17] have been developed for extraction of PAHs in the real sample matrices prior analysis by gas/liquid chromatography. However, ultrasonic-assisted extraction (UAE) method was considerably used as a green extraction method because of low toxic solvent consumption, high extraction efficiency, and less extraction time, especially for particulate matter or solid samples [22–24].

In this work, a fast and green extraction method (ultrasonic-assisted extraction) in combination with silica gel solid phase extraction clean-up of PAHs in the particulate matter samples was introduced. A capillary gas chromatography coupled with electron impact ionization tandem mass spectrometry (GC-EI-MS/MS) was used for detected and quantified of PAHs in particulate matter (PM2.5 and PM10) samples [25, 26]. GC-EI-MS/MS was used for this study because of the sensitivity and selectivity. The characteristics of the analytical method parameters such as limit of detection (LODs), limit of quantification (LOQs), linearity range, short- and long-term stability, and recovery of the extraction were investigated and presented. In addition, isotopic labelled internal standard was used in order to achieve the highest accuracy. The loss of the analytes during the sample preparation and other effects during GC-EI-MS/MS measurement were compensated by using isotopic labelled internal standards. The developed method was finally applied to determination of 16 PAHs in the particulate matter (PM2.5 and PM 10) samples collected in Hanoi. The emission source of the PAHs was quantified by using specific ratio of PAHs as proposal by Tobiszewski and Namieśnik [27]. “In addition, the toxicity of PAHs in the sampling area is calculated by toxic equivalency factor (TEQ) in which BaPs is the most toxic compound. The distribution of the PAHs in particulate matter sample was also investigated and implemented.

## 2. Materials and Methods

### 2.1. Chemicals and Reagents

The standard mixture of 16 PAHs (QTM PAH Mix, 2000 *µ*g mL^−1^ with 99.1% purity) including naphthalene (Naph), 2-bromonaphthalene (Br-Naph), acenaphthylene (Acy), acenaphthene (Ace), fluorene (Fln), phenanthrene (Phe), anthracene (Ant), fluoranthene (Flu), pyrene (Pyr), benz[a]anthracene (BaA), chrysene (Chr), benzo[b]fluoranthene (BbF), benzo[a]pyrene (BaP), indeno(1,2,3-cd)pyrene (IcdP), dibenz[a,h]anthracene (DahA), benzo[g,h,i]perylene (BghiP) and the isotopic labelled internal standard (EPA method 8270 internal standard mixture 2000 *µ*g mL^−1^ in dichloromethane) including naphthalene-D_8_ (Naph-D8), acenaphthene-D_10_ (Ace-D10), phenanthrene-D_10_ (Phe-D10), chrysene-D_12_ (Chr-D12), and perylene-D_12_ (Per-D12) were purchased from Sigma Aldrich (Singapore) and LGC (Germany), respectively. The working standard solutions were prepared at concentration levels 2, 5, 10, 25, 50, 100, 250, 500, 1000, and 2000 ng mL^−1^ with 200 ng mL^−1^ of all isotopic labelled internal standards in hexane (purity for GC-MS analysis, Merck, Singapore). Certificated reference material standard (TCL Polynuclear Aromatic hydrocarbon mix) with 95.9% purity was collected from Supelco (Singapore). Proficiency testing samples (International Sediment Exchange for Tests on Organic Contaminants) were taken from Wageningen Evaluating Programs for Analytical Laboratories (Netherlands). All of solvents such as hexane, acetone, dichloromethane (DCM), and methanol (MeOH) and the other chemicals such as Na_2_SO_4_, silica gel, and H_2_SO_4_ 98% with high purity grade were collected from Merck (Singapore). Anhydrous Na_2_SO_4_ was baked at 450°C for 3 hours and kept in an amber glass bottle in the desiccator.

### 2.2. Instruments

A gas chromatography (GC Trace 1310, Thermo Scientific, USA) including TriPlus RSH liquid autosampler coupled with an electron impact ionization-tandem mass spectrometry (Model TSQ 9000, Thermo Scientific, USA) was used for data acquisition. The sensitivity of the mass spectrometer was regularly checked by using perfluorotributylamine (PFTBA) as a tuning solution. A Thermo TG-5MS capillary column (30 m × 0.25 mm internal diameter × 0.25 *µ*m film thickness, 5% methylphenyl polysiloxane stationary phase) was used to separate 16 PAHs. Injection volume was set 1 *µ*L by liquid autosampler and using the splitless mode. Temperature of injector was kept at 280°C. Helium (99.999% purity) was used as carrier gas and constantly kept at 1.0 mL min^−1^ whole gas chromatographic separation time. 16 PAHs were separated by the program of temperature as follows: initial temperature was started from 80°C and kept for 3 minutes, raised linearly to 200°C at 15°C min^−1^, then increased continuously to 300°C at a rate 8°C min^−1^, and finally held for 5 minutes for conditioning GC column. The transfer line of the GC coupled with mass spectrometer was set at 300°C. Temperature of electron impact ionization source and quadrupole was kept constantly at 280°C and 150°C, respectively. Energy ionization was kept at 70 eV. PAHs were detected by positive EI-MS/MS in the multiple reaction monitoring (MRM) mode. Parameters of MRM of the PAHs and internal standard (precursor ions, product ions, and collision energy) are listed in Table S2 in the Supplementary Information.

### 2.3. Sample Preparation

#### 2.3.1. Sample Collection and Storage

**Figure 1:**
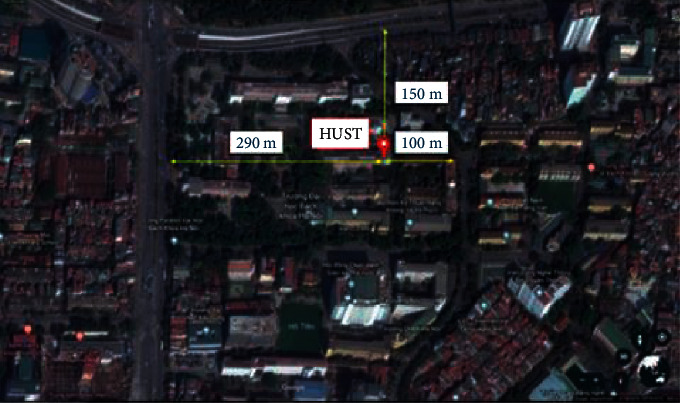
Sampling site of particulate matters for PAHs analysis.

Particulate matter samples were collected from the C4 building of Hanoi University of Science and Technology (HUST) in January 2018 (as shown in Figure 1). HUST is one of universities in the Hanoi center with crowded population and heavy traffic activities. Each sample was collected for 24 hours by using a sampler (MiniVol TAS, Air Metrics, Oregon, USA) with a low flow rate of sampling (5 L min^−1^). The sampler was placed above the ground approximate 1.5 m. Quartz fiber filter (47 mm, QH-A, Whatman, Merck, Singapore) was used for particulate matter sampling. Quartz fiber filter was baked at 450°C for at least 4 hours in order to remove all organic contaminants. Pre- and postsampling filter were weighted by microbalance (Model XPR U, Mettler Toledo, Switzerland). After sampling, particulate matter samples were wrapped in aluminum foil, packed in zipper plastic bag, transported to the laboratory, and stored in a refrigerator at 4°C until analysis. More information about sampling of the particulate matter is shown in Table S1 in the Supplementary Information.

#### 2.3.2. Sample Preparation

Particulate matter samples were extracted in a 15 mL prebaked centrifuge glass tube. Samples were spiked with 20 *µ*L (10 *µ*g mL^−1^) of isotopic labelled internal standard and stood at room temperature for at least 60 minutes for equilibrium. Samples were then extracted three times by ultrasonication for 5 minutes each time using VCX 130 PB ultrasonic probe (Sonics, Connecticut, USA), with 10 mL mixture of acetone/hexane (1/1, *v*/*v*), followed by centrifugation at 4500 ×g for 5 minutes. The extracts were combined and evaporated to 0.5 mL by gentle nitrogen flow and then exchanged to hexane and cleaned up by offline acidified silica gel solid phase extraction. The acidified silica cartridges were conditioned with 10 mL MeOH and then followed by 10 mL hexane at a flow rate of approximately 3 mL min^−1^. The extracts were transferred to the SPE cartridge and eluted with 12 mL mixture of hexane/dichloromethane (1/1: *v*/*v*). Eluent solutions were concentrated under a gentle stream of nitrogen to nearly dry and then refilled with exact 1 mL by hexane. These samples were subjected to analysis by GC-EI-MS/MS. The concentration of PAHs in the real samples was quantified by using isotopic labelled internal standard calibration curve.

### 2.4. Quality Assurance and Quality Control

Quality assurance and quality control (QA/QC) were conducted by performing laboratory blanks, sampling blanks, and recoveries of international sediment exchange for tests on organic contaminants samples (proficiency testing sample: PT sample). The laboratory blanks and sampling blanks were prepared and analyzed in the same manner as original samples. The relative standard deviation was controlled less than 15% for PAHs. In addition, PT sample was carried out independently 5 times to determine relative standard deviation following all analytical process. The recovery of individual compound in PT sample was compared with reported values. All glassware used for this study was baked at 450°C for at least three hours in order to remove all organic contaminants.

Besides, the stability of analytical signals was assessed by measuring a 2000 ng mL^−1^ standard solution, intraday and interday, and then calculating relative standard deviation of analytical signals. Limit of detection (LOD) was calculated as 3 times signal-to-noise ratio obtained from lowest matrix-matched samples. Meanwhile, the method limit of quantification (LOQ) was calculated by 10 times signal to noise at the lowest concentration matrix-matched samples [28–31]. The numbers of injection of blanks and QC samples in one analysis batch contained at least 20% of total numbers of injection.

### 2.5. Data Evaluation

All parameters of the GC-MS/MS system and quantification were controlled through Thermo Xcalibur software version 4.0 (Thermo Scientific, USA). Integration and data processing were performed quantitatively and qualitatively in Thermo Xcalibur software version 4.0 (Thermo Scientific, USA). All concentrations of PAHs in particulate matter samples were calculated and evaluated by using data analysis functions in Microsoft Excel 2020 (Microsoft, USA). All confident range of data was set 95% at 0.05 interval of confident. The presence of PAHs in real samples was identified by retention times, presenting of two transitions in MS/MS mode and relative peak area ratio of two transitions (from precursor ion to quantifier and confirmation ions) with a given relative of measurement uncertainty.

Due to the fact that the toxicity of PAHs is depended on the structure of individual compounds, therefore, toxicology of PAHs can be estimated by toxic equivalents (TEQ) based on the benzo[a]pyrene equivalent toxicity (BaPeq), in which BaP is defined as the most toxic compound and toxic equivalency factor (TEF) of this compound is unit [4]. According to average concentration of individual PAHs found in real samples, the BaP_eq_ can be calculated following the formula below:

(1) $$\begin{array}{c} {\text{BaP}}_{\text{eq}}=\sum \left({\text{BAP}}_{\text{eq}}\right)=\sum \left(C_{\text{PAH}i}\times {\text{TEF}}_{\text{PAH}i}\right), \end{array}$$

where *C*_PAH*i*_ is concentration of the individual PAH and TEF_PAH*i*_ is the respective toxic equivalency factor [32]. In addition, the emission source of PAHs was investigated and presented. In this work, the emission source of PAHs was assessed by specific compound ratios as proposed by Tobiszewski and Namieśnik [27].

## 3. Results and Discussion

### 3.1. Optimization of GC-EI-MS/MS Parameters for Analysis of PAHs

#### 3.1.1. Gas Chromatographic Separation

**Figure 2:**
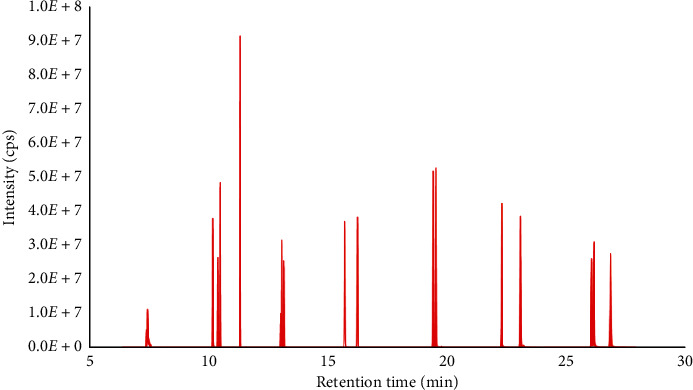
Total ion chromatogram of PAHs on Thermo TG 5 ms column.

In the most recent studies, gas chromatography capillary columns with nonpolar stationary phase have been selected for separation PAH compounds. In this work, a Thermo TG- 5MS capillary column (30 m × 0.25 mm i.d × 0.25 *µ*m thin film thickness) with (5%-phenyl)-methylpolysiloxane stationary phase was chosen for separation of PAHs. A total ion chromatogram (TIC) of PAHs at 2 *µ*g mL^−1^ standard solution is demonstrated in Figure 2. The temperature program, a critical factor in GG separation, could be optimized in order to achive the highest selectivity. Therefore, several different temperature programs were tested and the final temperature program was chosen as aforementioned. It could be clearly seen in Figure 2 that 16 PAHs target compounds have been baseline separated from each other with the selected temperature program. In addition, peak of all PAHs compounds was of good shape. Asymmetry factors of all PAHs ranged from to 0.91 (BghiP) to 1.45 (Naph). Retention times of individual PAH compounds are shown in Table 1.

#### 3.1.2. Optimization of Collision Energy (CE) for Individual Compounds in MS/MS Mode

Another critical parameter in GC-EI-MS/MS measurement, collision energy, was investigated in order to get the highest sensitivity and selectivity of measurement. For investigation of the collision energy in MS/MS mode, two transitions of each analyte in MS/MS mode including isotopic labelled internal standards were chosen for measurement. A given concentration of PAHs was injected at the same chromatographic condition (at the same temperature program) but at the different of the collision energy (from 10 to 40 eV with 5 eV step) and then the normalized peak area was calculated and plotted as a function of the collision energy.  Figure S2 in the Supplementary Materials shows the normalized peak area of all MS/MS transitions depending on the collision energy.

As shown in Figure S2, the highest normalized peak of transitions was selected for quantification and confirmation of PAHs. For example, collision energies of ACE were 15 eV and 30 eV for transitions from precursor ion to quantifier ion and from precursor ion to qualifier ion, respectively. The selection of collision energy of transitions of isotopic labelled internal standards was performed in the same manner of native standard. The optimum collision energy of MS/MS transitions was chosen and is listed in Table 1. For summary, the optimized temperature program and collision energy was selected for further experiments as shown in the Supplementary Information.

### 3.2. Analytical Characteristic of the Developed Method

#### 3.2.1. Linearity Range, Limit of Detection (LOD), and Limit of Quantification (LOQ)

Eight independent standard solutions at levels of 5, 10, 50, 100, 200, 500, 1000, and 2000 ng mL^−1^ for all target analytes with 200 ng mL^−1^ isotopic labelled internal standards were prepared by dilution stock solution in *n*-hexane and then injected into the GC-EI-MS/MS systems. The calibration curves were built based on the ratio of peak area between native standard and associate isotopic labelled internal standards with concentration of target compounds. The calibration curve equations with correlation coefficients of 16 PAHs are listed in Table 2. As clearly shown in Table 2, the excellent correlation coefficient between peak area ratio of analytical signal and concentration of analytes was achieved (*R*^2^ ≥ 0.9990 for all target analytes). Also, it can be clearly seen from Table 2 that LODs and LOQs of the developed method were calculated. LODs and LOQs values ranged from 0.001 ng m^−3^ (Br-Naph) to 0.276 ng m^−3^ (Fln) and 0.004 ng m^−3^ (Br-Naph) to 0.829 ng m^−3^ (Fln).

#### 3.2.2. Stability of the Analytical Signal

Short-term and long-term stabilities of the analytical signal play a critical role in measurement of uncertainty and robustness of the developed analytical method. For assessment stability of the analytical method, two sets of five solutions containing all target analytes at a concentration of 2 *µ*g mL^−1^ were prepared in solvent. This solution was injected on the GC-EI-MS/MS at the optimum operating conditions. The short-term and long-term stabilities were performed for 1 day and 2 days of continuous measurement, respectively. The relative standard deviations of peak area ratio between native standard and isotopic labelled internal standard of all PAHs are shown in Table 3. As can be clearly seen from Table 3, the excellent repeatability of the analytical signals was achieved for both short term and long term with RSD of the ratio of peak area between native and isotopic labelled internal standards being below 2.6% and 3.7%, respectively. All RSD values of the analytical signal were lower than the acceptable value according to Horwitz [33].

#### 3.2.3. Extraction Recovery of PAHs

In this work, extraction recovery of individual compound was evaluated through analyzing PT sample. The experiment was replicated five times following aforementioned analytical procedure. As clearly shown in Table 3, the mean recoveries were in the range from 79.3% (Chr) to 109.8% (IcdP).

The recovery of ultrasonic-assisted extraction and clean-up procedure fell in the acceptable range according to AOAC guidelines [34]. In addition, the loss of the analytes during sample preparation steps and fluctuation of analytical signal during measurement time could be compensated by using isotopic labelled internal standards.

### 3.3. Analysis of Real Samples

#### 3.3.1. PAH Concentration in Real Samples

**Figure 3:**
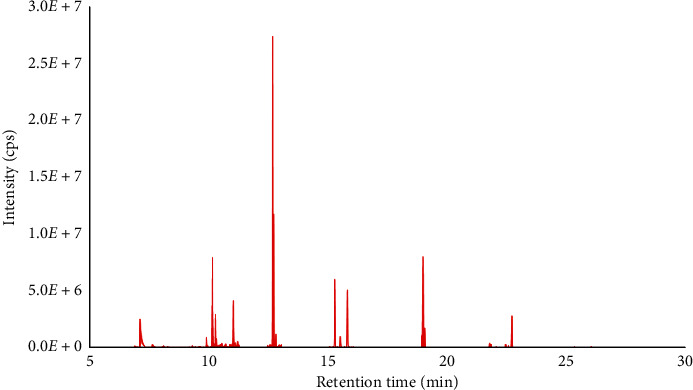
Total selected ion chromatogram of PAHs in real sample.

For application, the validated method was used for analysis of 16 PAHs in ten investigated particulate matter samples collected from Hanoi, Vietnam. The ultrasonic-assisted extraction and SPE cleaning-up were used for sample preparation. The concentration of PAHs in the real samples was analyzed by GC-EI-MS/MS. Total selected ion chromatogram of PAHs found in the real sample is demonstrated in Figure 3. The mean concentration of PAHs in the real samples is listed in Table 4. From that, all of target compounds were found in the real samples, with the total concentration of PAHs from 226.3 ng m^−3^ to 727.3 ng m^−3^ and lower than that in 2007 (290–1300 ng m^−3^). On the other hand, a study in Mexico has given information about PAHs content approximately in the range from 50 ng m^−3^ to 910 ng m^−3^. In another study performed in the spring in Beijing (China), the total concentration of PAH was approximately 4-5 times lower than that in this work. The total concentration of PAHs in PM 10 (727.3 ng m^−3^) was observed approximately 1.5 times higher than that in PM 2.5 (461.7 ng m^−3^) in terms of comparison between two particulate matter fractions. Considering BaP as the most toxic compound among 16 PAH compounds, it was found at low level (from 0.8 ng m^−3^ to 3.4 ng m^−3^) in all investigated samples. Meanwhile, it fluctuated from 4 ng m^−3^ to 69 ng m^−3^ in industrial and traffic areas in Germany [35]. Total concentration of low-molecular weight PAHs was from 202.7 ng m^−3^ to 705.8 ng m^−3^ and high-molecular weight was from 20.7 ng m^−3^ to 71.1 ng m^−3^. In addition, concentration of PAH in PM 10 sample was higher than that in PM 2.5 on the day of 18^th^, 19^th^, 21^st^, and 23^rd^/01/2018. However, this trend is opposite on January 20^th^, 2018. The weather on that day was wetter than on the others which may be the main reason. The relationship of PAHs concentration with temperature, relative humidity, and other meteorological parameters should be considered for this situation.

**Figure 4:**
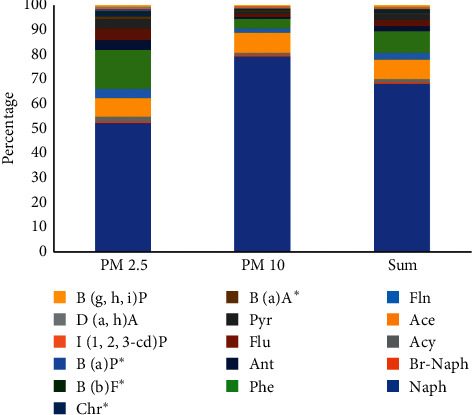
Distribution of individual PAHs in some kinds of particulate matter samples.

Figure 4 demonstrates the distribution of individual PAH in PM 2.5 and PM 10 samples collected from an area of Hanoi University of Science and Technology (HUST). There was a similar trend among 16 PAHs in the two fraction samples. Naphthalene was found at the highest frequency in all analyzed samples, accounting for 52.2% and 79.2% in total of PM 2.5 and PM 10, respectively. Considerably, seven PAHs (BaA, Chr, BbF, BaP, IcdP, DahA, BghiP) which are well-known as most toxic compound according to IARC were found at the lowest concentration in both of particulate matter partitions (<1%). This reason should explain the fact that the molecular weight of PAH is lower, and its evaporation becomes more and more easy. In spite of the fact that heavy molecular weight compounds were observed at low concentration, air pollution at the studied area was clear. The exposure to these compounds at high concentration through particulate matter can cause negative impact on human health. Moreover, the finding of naphthalene in all samples at the highest frequency also has warned us about the pollution of this compound in the sampling site. A remarkable aspect is that there was a similar tendency of distribution in both types of particle for PAHs with high-molecular weight (six fused rings in the structure) such as IcdP, DahA, and BghiP. In contrast, PAHs belonging to the low-molecular weight (four or fewer fused rings) were different distribution in PM 10 and PM 2.5. It could attribute to several factors [35, 36] such as temperature, humidity, molecular weight, and human activities.

#### 3.3.2. Assessment of PAH Toxicology through TEQ Value

However, all PAHs do not show the same toxicity. Toxicity of these compounds depends on their structure and substituted groups. Therefore, the toxicity equivalency factor (TEF) approach was used to convert to benzo(a)pyrene-equivalent (BaPeq) toxicity. The mean concentration of PAHs in two particulate matter fractions (PM 2.5 and PM 10) was used for computation of equivalent toxicity. Using equation (1), the equivalent toxicology of PAHs in two particulate matter fractions was calculated and is presented in Table 4.

Table 4 gives information about TEQ of PAHs in PM 2.5 (4.66 ng m^−3^) higher than in PM 10 (3.68 ng m^−3^). This results continuously warn us about air pollution, especially in the fine-fraction particulate matter, in Hanoi, because particulate matter with small particle size like PM 2.5 can easily migrate to human body through breathing and then lead to deleterious impact on human health, especially lung cancer and genetic mutations.

#### 3.3.3. Emission Source of the PAHs

Estimation of PAH emission source also plays an important role in controlling the quality of air in Hanoi. Nowadays, there are two popular ways used in assessment of emission source, such as using principal component analysis (PCA) statistical tool and determining the specific ratios between several PAH isomers [36]. In this work, the second way was used for assessment of emission source of PAHs. The ratio of Ant/(Phe + Ant) > 0.1, especially Flu/(Pyr + Flu) > 0.5 and BaA/(BaA + Chr) > 0.35 demonstrated that the PAH emission in studied area mostly resulted from organic incomplete combustion such as coal, wood, or firewood in daily life activities. Besides, the values of BaP/BghiP > 0.5 have shown that there was a contribution of traffic activities in PAHs emission into environment, especially vehicles running by gasoline and oil (IcdP/(IcdP + BghiP) = 0.5). All ratios of specific PAHs in the analyzed particulate matter samples were calculated. By using these ratios, the emission sources of the PAHs in the particulate matter samples in this study mostly come from transportation traffic activity, for instance, from car and motorbike cycle activity in Hanoi.

## 4. Conclusion

A green extraction in combination with GC-EI-MS/MS method was successfully developed for analysis of 16 priority PAHs in particulate matter fractions (PM 10 and PM 2.5) collected from Hanoi, Vietnam. All critical parameters of analytical method such as linearity range, coefficients, LOD, LOQ, and recovery have been investigated and implemented. The developed method was applied to determine PAHs in some real samples collected from Hanoi, Vietnam. The experimental results have shown that the main sources of PAHs in these samples come from fossil fuel burning such as traffic and household activities. The results also warned about PAH pollution in the studied area, especially originating from traffic and daily-life activities. For the next steps, variance of PAHs concentration depending on the different particulate matter fractions (from nanometer to micrometer diameter), seasons, and weather will be addressed and presented. In addition, human exposure to these compounds through outdoor activities will be investigated and implemented.

## Figures and Tables

**Table 1:** Retention times, MS/MS transitions, and collision energy of 16 PAHs and isotopic labelled internal standards in MS/MS measurement.

Name	RT (min)	Quantifier	CE	Qualifier	CE
Naph	7.16	128.08 ⟶ 102.06	15	128.08 ⟶ 127.09	15
*Naph-D*8	7.13	136.13 ⟶ 136.13	10		
Acy	9.91	152.08 ⟶ 151.10	15	152.08 ⟶ 150.08	30
Br-Naph	10.12	208.00 ⟶ 127.08	20	208.00 ⟶ 205.99	25
Ace	10.21	153.11 ⟶ 152.11	15	154.12 ⟶ 152.12	30
*Ace-D*10	10.16	162.16 ⟶ 162.16	10	164.17 ⟶ 162.16	10
Fln	11.04	166.10 ⟶ 165.10	15	166.10 ⟶ 163.08	40
Phe	12.74	178.11 ⟶ 152.09	20	178.11 ⟶ 176.11	25
*Phe-D*10	12.72	188.17 ⟶ 188.17	10	188.17 ⟶ 160.14	20
Ant	12.82	178.11 ⟶ 152.08	20	178.11 ⟶ 176.10	25
Flu	15.3	202.08 ⟶ 200.10	30	202.08 ⟶ 201.10	20
Pyr	15.83	202.11 ⟶ 200.10	35	202.11 ⟶ 201.11	20
B(a)A	18.97	228.11 ⟶ 226.12	30	228.11 ⟶ 224.09	40
Chr	19.08	228.12 ⟶ 226.11	30	228.12 ⟶ 224.09	40
*Chr-D*12	19.01	240.19 ⟶ 236.18	30		
BbF	21.81	252.12 ⟶ 250.12	30	252.12 ⟶ 248.10	40
BaP	22.59	252.11 ⟶ 250.12	35	252.11 ⟶ 248.09	40
*Per-D*12	22.74	264.19 ⟶ 260.19	40		
IcdP	25.37	276.15 ⟶ 274.12	40	274.12 ⟶ 272.08	35
DahA	25.46	278.15 ⟶ 276.12	35	276.12 ⟶ 274.10	30
BghiP	26.09	276.14 ⟶ 274.10	40		

**Table 2:** Analytical figure of merits of PAHs on the GC-EI-MS/MS.

Compounds	RT (min)	Calibration curves	*R* ^2^	LODs (ng m^−3^)	LOQs (ng m^−3^)
Naph	7.16	*Y* = (0.00069 ± 2.5*E*-6)*X* + (0.00343 ± 0.002)	1.0000	0.002	0.006
Br-Naph	10.12	*Y* = (0.00097 ± 4.56*E*-6)*X*—(0.00894 ± 0.004)	0.9994	0.001	0.004
Acy	9.91	*Y* = (0.00261 ± 1.38*E*-5)*X*—(0.01670 ± 0.012)	0.9998	0.080	0.241
Ace	10.21	*Y* = (0.00361 ± 1.95*E*-5)*X*—(0.01213 ± 0.017)	0.9998	0.072	0.216
Fln	11.04	*Y* = (0.00627 ± 2.31E-5)*X*—(0.03750 ± 0.020)	0.9998	0.276	0.829
Phe	12.74	*Y* = (0.00095 ± 6.64*E*-6)*X*—(0.00618 ± 0.006)	0.9999	0.004	0.012
Ant	12.82	*Y* = (0.00081 ± 7.14*E*-6)*X*—(0.00815 ± 0.006)	0.9998	0.010	0.030
Flu	15.3	*Y* = (0.00144 ± 1.07*E-*5)*X*—(0.01369 ± 0.009)	0.9998	0.008	0.024
Pyr	15.83	*Y* = (0.00159 ± 1.31*E*-5)*X*—(0.01590 ± 0.011)	0.9998	0.005	0.014
BaA	18.97	*Y* = (0.00519 ± 3.55*E*-5)*X*—(0.03670 ± 0.031)	0.9998	0.002	0.007
Chr	19.08	*Y* = (0.00525 ± 3.13*E*-5)*X*—(0.03239 ± 0.027)	0.9998	0.004	0.013
BbF	21.81	*Y* = (0.00932 ± 4.04*E*-5)*X*—(0.01775 ± 0.035)	0.9997	0.144	0.433
BaP	22.59	*Y* = (0.00742 ± 3.34*E*-5)*X*—(0.01894 ± 0.029)	0.9999	0.002	0.007
IcdP	25.37	*Y* = (0.00541 ± 3.15*E*-5)*X*—(0.06213 ± 0.027)	0.9995	0.041	0.123
DahA	25.46	*Y* = (0.00677 ± 4.14*E*-5)*X*—(0.06589 ± 0.036)	0.9995	0.202	0.605
BghiP	26.09	*Y* = (0.00562 ± 4.23*E*-5)*X*—(0.04773 ± 0.037)	0.9990	0.012	0.035

**Table 3:** Proficiency testing sample based recovery and stability of analytical signal in GC-EI-MS/MS analysis of PAHs.

No.	Compounds	Reported conc. (ng g^−1^)	Exp. conc. (ng g^−1^) (*n* = 5)	RE ± RSD (%) (*n* = 5)	Stability of analytical signal, RSD (%)	
					Short-term	Long-term
1	Naph	467 ± 230.9	466 ± 185	99.7 ± 4.0	0.3	0.4
2	Acy	390.4 ± 127.0	386.2 ± 41.4	98.9 ± 10.7	1.1	1.4
3	Ace	185.1 ± 34.1	171.6 ± 17.9	92.7 ± 10.4	1.4	1.8
4	Fln	201.2 ± 49.6	160.2 ± 1.8	79.6 ± 3.6	1.3	1.6
5	Phe	2688 ± 395	2546.2 ± 33.3	94.7 ± 1.3	0.3	0.9
6	Ant	663.1 ± 173.9	693.6 ± 23.9	104.5 ± 3.5	0.6	0.7
7	Flu	6753 ± 972	6776.7 ± 555	100.4 ± 6.3	1.0	1.1
8	Pyr	5387 ± 568	5877.1 ± 529	109.1 ± 9.0	0.2	0.9
9	BaA	3607 ± 697	3656.4 ± 197	101.4 ± 5.0	0.8	1.2
10	Chr	3822 ± 413	3673.4 ± 49.8	79.3 ± 1.4	1.1	1.5
11	BbF	4246 ± 887	3367.3 ± 279	92.8 ± 8.3	1.5	2.2
12	BaP	3345 ± 603	2815.4 ± 164	84.2 ± 5.8	0.4	1.5
13	IcdP	2703 ± 531	2968.4 ± 426	109.8 ± 4.3	2.3	3.4
14	DahA	647.2 ± 219	645.3 ± 72.9	99.7 ± 9.3	2.6	3.7
15	BghiP	2703 ± 488	2242.1 ± 41.4	82.9 ± 1.9	2.4	3.33

**Table 4:** TEF of 16 PAHs and TEQ of PAHs in the investigated samples.

PAH	TEF	PM 2.5		PM10	
		*C*_PAH*i*_ (ng m^−3^)	BaP_eq*i*_ (ng m^−3^)	*C*_PAH*i*_ (ng m^−3^)	BaP_eq*i*_ (ng m^−3^)
Naph	0.001	179.30	0.18	389.70	0.390
Br-Naph	0.001	1.90	0.00	3.09	0.003
Acy	0.001	6.71	0.01	3.60	0.004
Ace	0.001	26.35	0.03	40.58	0.041
Fln	0.001	12.61	0.01	8.78	0.009
Phe	0.001	53.54	0.05	19.92	0.020
Ant	0.01	14.29	0.14	2.68	0.027
Flu	0.001	16.44	0.02	6.22	0.006
Pyr	0.001	13.07	0.01	5.23	0.005
B(a)A ^*∗*^	0.01	2.97	0.03	1.44	0.014
Chr ^*∗*^	0.01	4.83	0.05	1.97	0.020
B(b)F ^*∗*^	0.1	3.19	0.32	1.98	0.198
B(a)P ^*∗*^	1	2.31	2.31	1.48	1.484
I(1,2,3-cd)P	0.1	2.20	0.22	2.07	0.207
D(a,h)A	1	1.26	1.26	1.24	1.237
B(g,h,i)P	0.01	2.22	0.02	2.14	0.021
ΣBaP eq			4.66		3.68

## Data Availability

The data used to support the findings of this study are included within the article.

## Supplementary Materials

Supplementary Materials Associated with this manuscript is Supplementary Information that contains 2 tables and 2 figures listed as follows: Table S1: information of samples collected from Hanoi University of Science and Technology. Table S2: MS/MS transitions and collision energy of PAHs and isotopic labelled internal standards. Figure S1: structures of PAHs and isotopic labelled internal standards. Figure S2: collision energy of 16 PAHs and isotopic labelled internal standards.
