# Supplementary material for: Analysis of Polycyclic Aromatic Hydrocarbon in Airborne Particulate Matter Samples by Gas Chromatography in Combination with Tandem Mass Spectrometry (GC-MS/MS)
Source: J Anal Methods Chem. 2021 May 26;2021:6641326. doi: 10.1155/2021/6641326 (PMC8175174; doi:10.1155/2021/6641326)
Supplement: Supplementary Materials — Associated with this manuscript is Supplementary Information that contains 2 tables and 2 figures listed as follows: Table S1: information of samples collected from Hanoi University of Science and Technology. Table S2: MS/MS transitions and collision energy of PAHs and isotopic labelled internal standards. Figure S1: structures of PAHs and isotopic labelled internal standards. Figure S2: collision energy of 16 PAHs and isotopic labelled internal standards. [file 6641326.f1.docx]

***Supplementary Information***

**Analysis of polycyclic aromatic hydrocarbon in particulate matter samples by gas chromatography in combination with tandem mass spectrometry (GC-MS/MS)**

Nam Vu-Duc^1^, Lan Anh Phung Thi^2^,Thuy Le-Minh^1^, Lan-Anh Nguyen^1^, Huong Nguyen-Thi^1^, Loan-Ha Pham-Thi^4^, Van-Anh Doan-Thi^1^, Huong Le-Quang^1^, Hung Nguyen-Xuan^1^, Thao Thi Nguyen^2^, Phuong Thanh Nguyen^3^, Dinh Binh Chu^4*^

1. Center for Research and Technology Transfer, Vietnam Academic of Science and Technology, 18 Hoang Quoc Viet, Hanoi 100000, Vietnam

2. School of Environmental Science and Technology, Hanoi University of Science and Technology, No1 Dai Co Viet, Hanoi 100000, Viet Nam

3. FPT University, Hoa Lac High Tech Park, Km 29 Thang Long Boulevard, Thach That, Hanoi 100000, Viet Nam

4. Department of Analytical Chemistry, School of Chemical Engineering, Hanoi University of Science and Technology, No1 Dai Co Viet Road, Hanoi 100000, Vietnam

**Sample collection and preparation**

Dust samples were collected from the C4 building of Hanoi University of Science and Technology (HUST) during January 2018 using a MiniVol TAS sampler. The information of the samples was listed in the Tables S1. Quartz fiber filter (47 mm, QH-A, Whatman, Merck, Singapore )was used for particulate mater sampling. Quartz fiber filter was baked at 450^o^C at least 4 hours in order to remove all organic contaminants. Pre- and post- sampling filter were weighted by microbalance (Model XPR U, Mettler Toledo, Switzerland).

Samples was spiked internal standard and then extracted by using three times of 10 mL mixture of acetone /n–hexane (1/1: v/v) in baked centrifuge glass tube (15 mL, Thomas Scientific, USA) by using VCX 130 PB ultrasonic processor. Organic solvent was separated by centrifugation (Model EBA 200, Hettich, Germany). All extracted solvent was combined and cleanup by manually acidic silica gel solid phase extraction. The acidic silica gel was prepared by pack 2 g of baked Na_2_SO_4_, 4 g of silica gel, 3 g of silica gel 40% H_2_SO_4_, 3 g of silica gel 20% H_2_SO_4_, then 2 g of baked Na_2_SO_4_ in the pre-clean chromatographic glass tube. SPE column was conditioned by 100 mL n-hexane. Sample was loaded on the SPE column and eluted by mixture of hexane – dichloromethane ((1/1: v/v)). Eluted solvent was collected in the pre-cleaned graduated glass tube and concentrated by using gent stream of nitrogen until nearly dryness. The sample was refilled with n-hexane until reaching exactly 1 mL. Samples were subjected to analysis by GC-EI- MS/MS

**GC-EI-MS/MS operating conditions for analysis of PAHs**

| **GC operating conditions** | **Trace GC 1310 with TriPlus RSH autosampler** |
| --- | --- |
| Column | Thermo TG 5MS (30 m x 0.25 mm internal diameter x 0.25 µm film thickness, 5 % methylphenyl polysiloxane stationary phase) |
| Carrier gas | Helium 99.9999% |
| Flow of carrier gas | 1 mL min ^-1,^ constant flow mode |
| Temperature program | 80 ^o^C and kept for 3 minutes |
|  | 15 ^o^C min^-1^ to 200 ^o^C |
|  | 8 ^o^C min^-1^ to 300 ^o^C |
|  | 300 ^o^C hold for 5 minutes |
| Temperature of injector | 280 ^o^C |
| Injection volume | 1 µL |
| Injection mode | spitless |
| Temperature of transferline | 300 ^o^C |
| **MS operating conditions** | **TSQ 9000 mass spectrometer** |
| Ion source temperature | 280 ^o^C |
| Temperature of quadrupole | 150 ^o^C |
| Ionization mode | electron impact ionization |
| Polarity | Positive |
| Ionization energy | 70 eV |
| Measurement mode | MRM mode |
| Collision gas | Argon 99.9999 % |
| Mass resolution | Q1, Q3 normal (0.7 Da at FWHM) |

Table S1: Information of samples that collect from Hanoi University of Science and Technology

| **Airborne particle** | **Samples** | **Time of collection** | **Weight of sample (g)** | **Volume of sample**  **(m^3^)** |
| --- | --- | --- | --- | --- |
| PM 2.5 | T13 | 18/01/2018 | 0.000702 | 7.1 |
|  | T50 | 19/01/2018 | 0.000472 | 5.4 |
|  | T11 | 20/01/2018 | 0.000758 | 7.1 |
|  | T03 | 21/01/2018 | 0.000648 | 7.1 |
|  | T06 | 23/01/2018 | 0.000503 | 7.1 |
| PM 10 | T09 | 18/01/2018 | 0.000956 | 7.1 |
|  | T58 | 19/01/2018 | 0.000863 | 5.4 |
|  | T14 | 20/01/2018 | 0.000950 | 7.1 |
|  | T08 | 21/01/2018 | 0.000869 | 7.1 |
|  | T10 | 23/01/2018 | 0.000674 | 7.1 |

Table S2. MS/MS transitions and collision energy of PAHs and internal standards

| **No** | **Analyte** | **MS/MS transition** | **CE (eV)** | **Internal standard** |
| --- | --- | --- | --- | --- |
| 1 | Naph | 128.1 ⇒ 127.1 | 15 | Naph-D8 |
|  |  | **128.1** ⇒ **102.1** | 15 |  |
| 2 | Naph-D8 | **136.1** ⇒ **136.1** | 10 |  |
| 3 | Acy | 152.1 ⇒ 150.1 | 30 | Ace-D10 |
|  |  | **152.1** ⇒ **151.1** | 15 |  |
| 4 | Ace | 154.1 ⇒ 152.1 | 30 | Ace-D10 |
|  |  | **153.1** ⇒ **152.1** | 15 |  |
| 5 | Ace-D10 | 164.2 ⇒ 162.2 | 10 |  |
|  |  | **162.2** ⇒ **162.2** | 10 |  |
| 6 | BrNA | 208.0 ⇒ 206.0 | 25 | Naph-D8 |
|  |  | **208.0** ⇒ **127.1** | 20 |  |
| 7 | Fln | **166.1** ⇒ **165.1** | 15 | Ace-D10 |
|  |  | 166.1 ⇒ 163.1 | 40 |  |
| 8 | Phe | 178.1 ⇒ 176.1 | 25 | Phe-D10 |
|  |  | **178.1** ⇒ **152.1** | 20 |  |
| 9 | Phe-D10 | 188.2 ⇒ 160.1 | 20 |  |
|  |  | **188.2** ⇒ **188.2** | 10 |  |
| 10 | Ant | 178.1 ⇒ 176.1 | 25 | Phe-D10 |
|  |  | **178.1** ⇒ **152.1** | 20 |  |
| 11 | Flu | **202.1** ⇒ **200.1** | 30 |  |
|  |  | 202.1 ⇒ 201.1 | 20 |  |
| 12 | Pyr | **202.1** ⇒ **200.1** | 35 |  |
|  |  | 202.1 ⇒ 201.1 | 20 |  |
| 13 | BaA | **228.1** ⇒ **226.1** | 30 | Chr-D12 |
|  |  | 228.1 ⇒ 224.1 | 40 |  |
| 14 | Chr | **228.1** ⇒ **226.1** | 30 |  |
|  |  | 228.1 ⇒ 224.1 | 40 |  |
| 15 | Chr-D12 | **240.2** ⇒ **236.2** | 30 |  |
| 16 | BbF | **252.1** ⇒ **250.1** | 30 | Per-D12 |
|  |  | 252.1 ⇒ 248.1 | 40 |  |
| 17 | BaP | **252.1** ⇒ **250.1** | 35 |  |
|  |  | 252.1 ⇒ 248.1 | 40 |  |
| 18 | IcdP | **276.2** ⇒ **274.1** | 40 |  |
|  |  | 274.1 ⇒ 272.1 | 35 |  |
| 19 | DahA | **278.2** ⇒ **276.1** | 35 |  |
|  |  | 276.1 ⇒ 274.1 | 30 |  |
| 20 | BghiP | **276.1** ⇒ **274.1** | 40 |  |
| 21 | Per-D12 | **264.2** ⇒ **260.2** | 40 |  |

*Note: transitions in bold letter were use for quantification and the other were used for conformation in combination with relative ratio between two transitions of each analyte and retention time on the GC column.*

|  |  |  |
| --- | --- | --- |
| Acenaphthene (Ace) | D10-Acenaphthene (Ace-D10) | Pyrene (Pyr) |
|  |  |  |
| Benz[a]anthracene (BaA) | 2-Bromonaphthalene (Br-Naph) | Chrysene (Chr) |
|  |  |  |
| D12-Chrysene (Chr-D12) | Florene (Fln) | Phenanthrene (Phe) |
|  |  |  |
| D10-Phenanthrene (Phe-D10) | Benzo[b]fluoranthene (BbF) | Benzo[a]pyrene (BaP) |
|  |  |  |
| Anthracene (Ant) | Indeno(1,2,3-cd)pyrene (IcdP) | Fluoranthene (Flu) |
|  |  |  |
| Dibenz[a,h]anthracene (DahA) | Benzo[g,h,i]perylene (BghiP) | D12-Perylene (Per-D12) |
|  |  |  |
| Naphthalene (Naph) | D8-Naphthalene (Naph-D10) | Acenaphthylene (Acy) |
| Figure S1. Structures of PAHs and isotopic labelled internal standards | | |

| 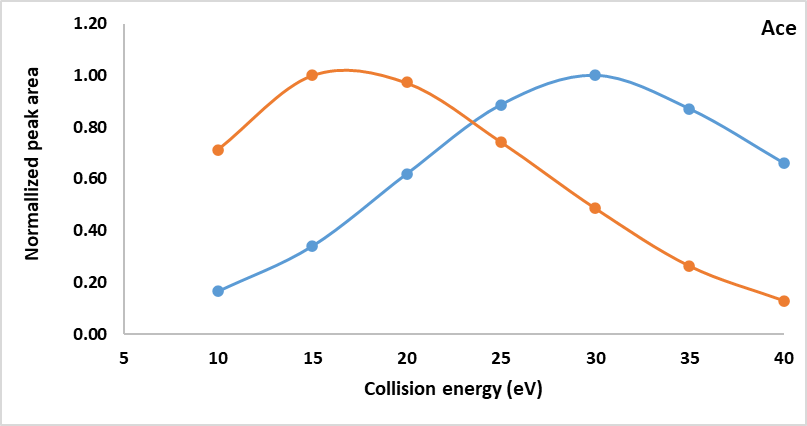 | 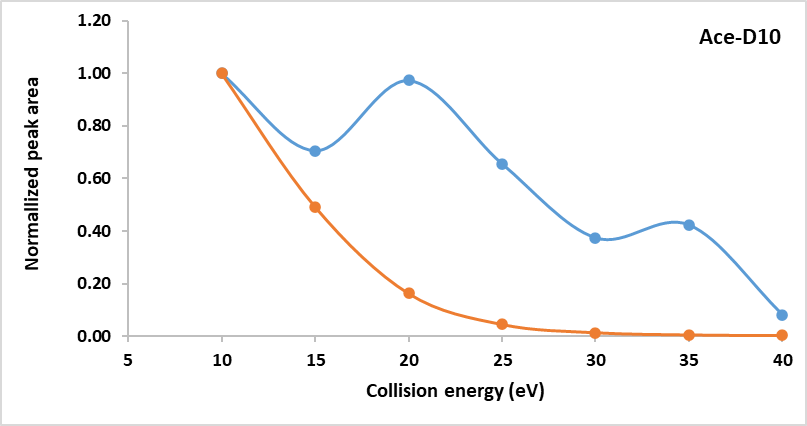 | 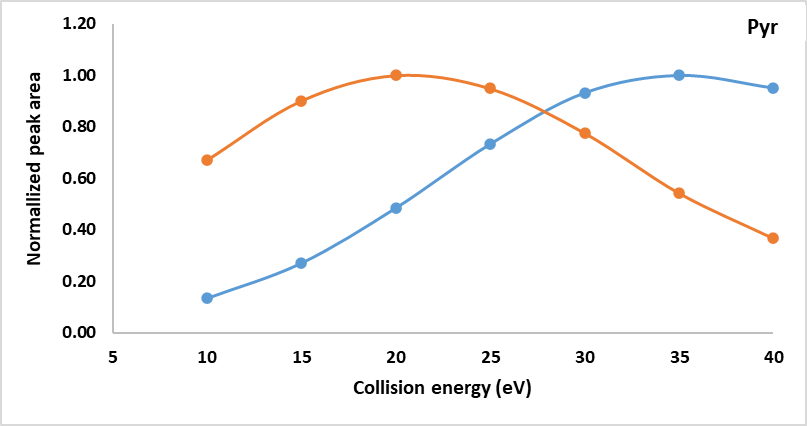 |
| --- | --- | --- |
| 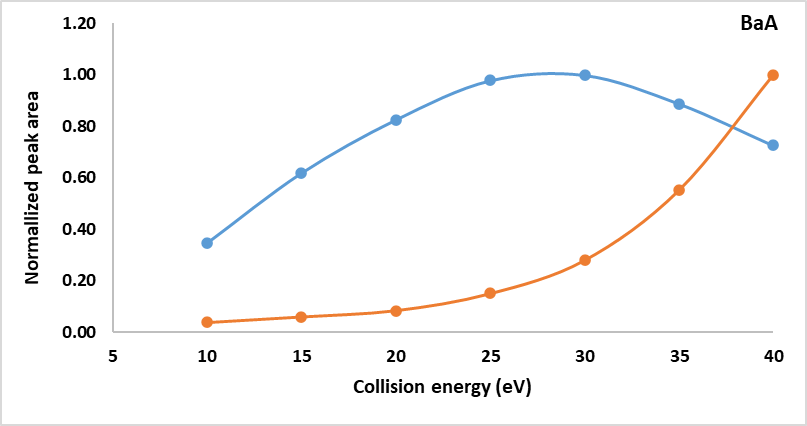 | 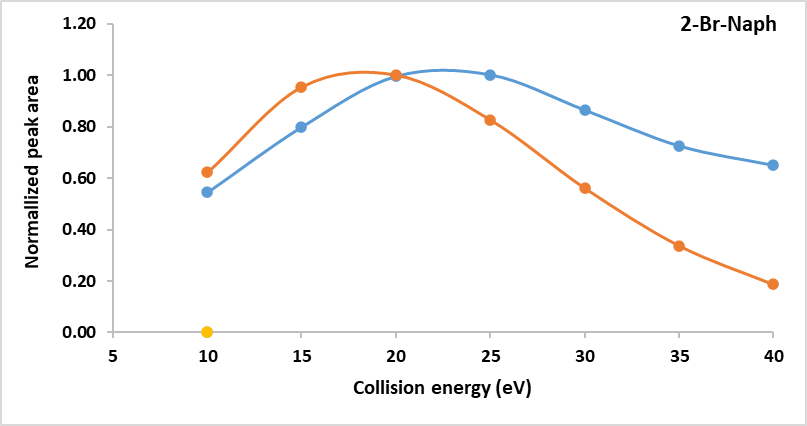 | 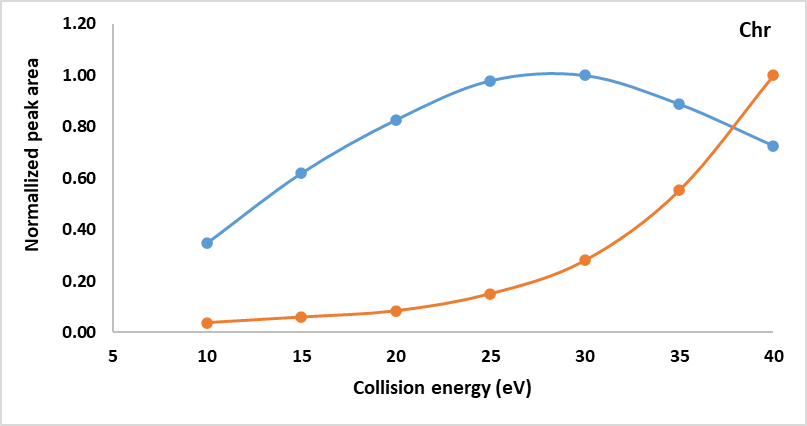 |
| 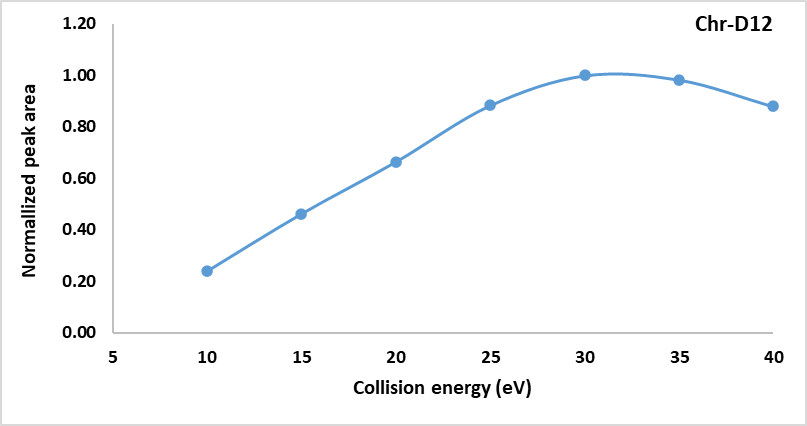 | 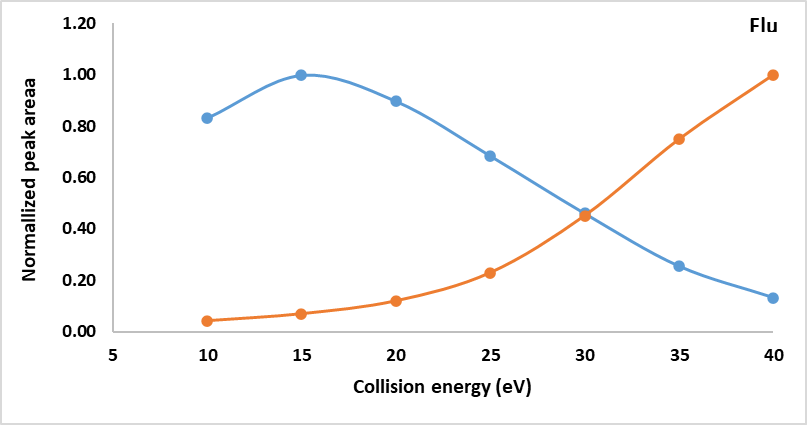 | 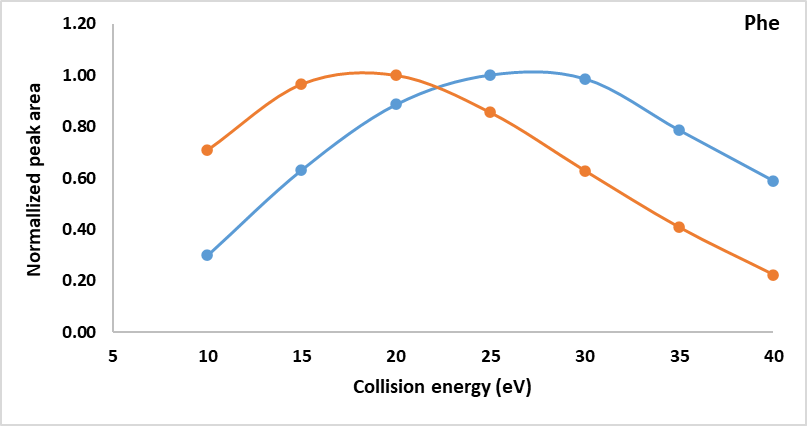 |
| 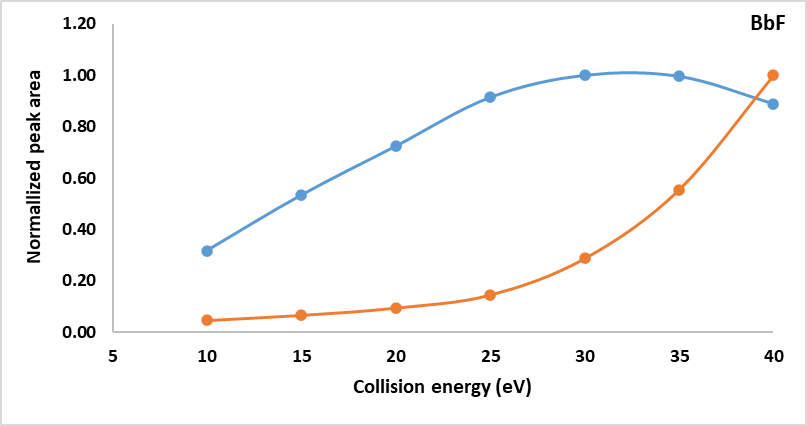 | 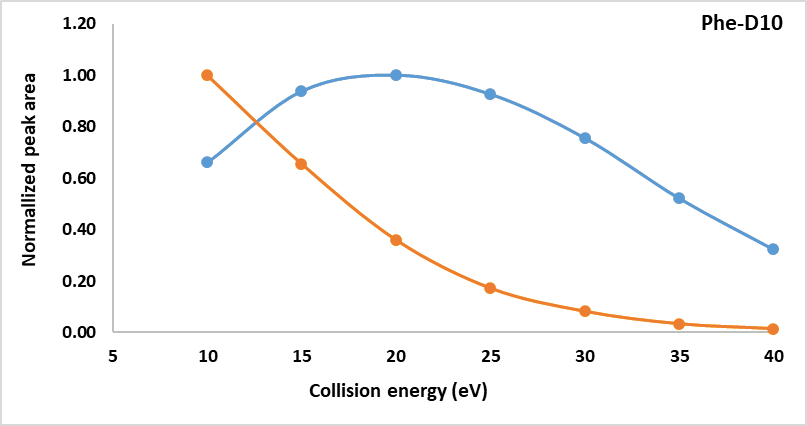 | 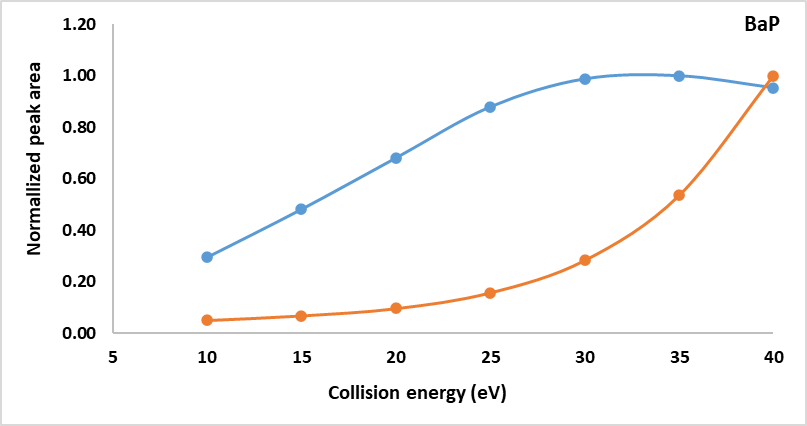 |
| 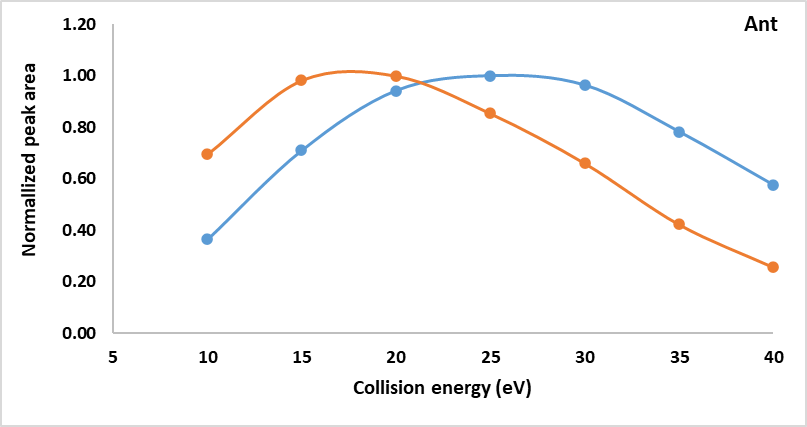 | 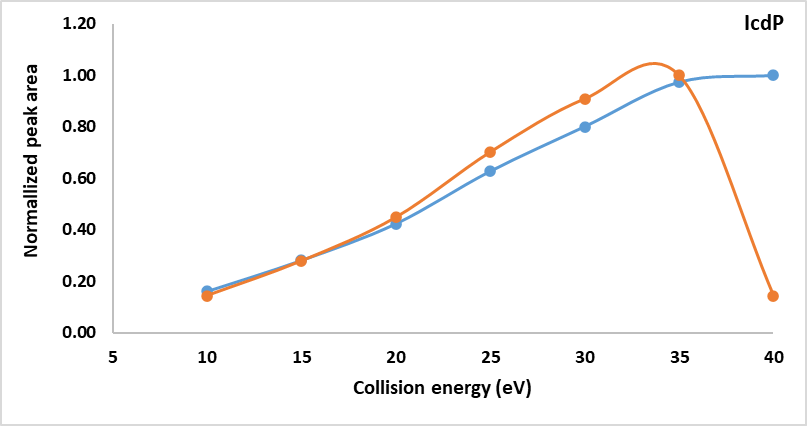 | 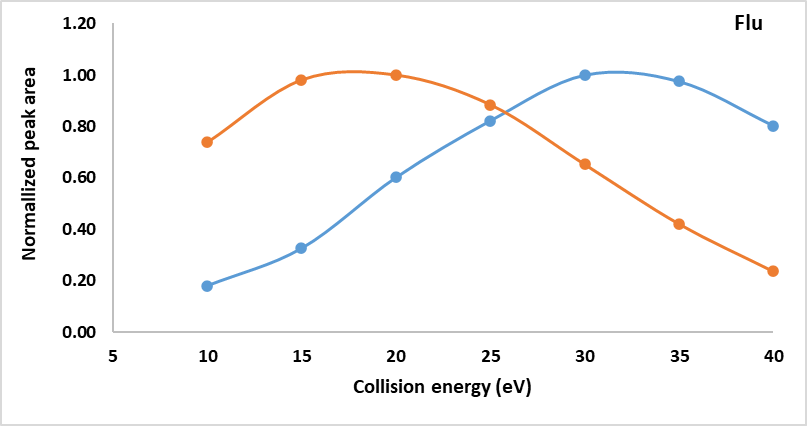 |
| 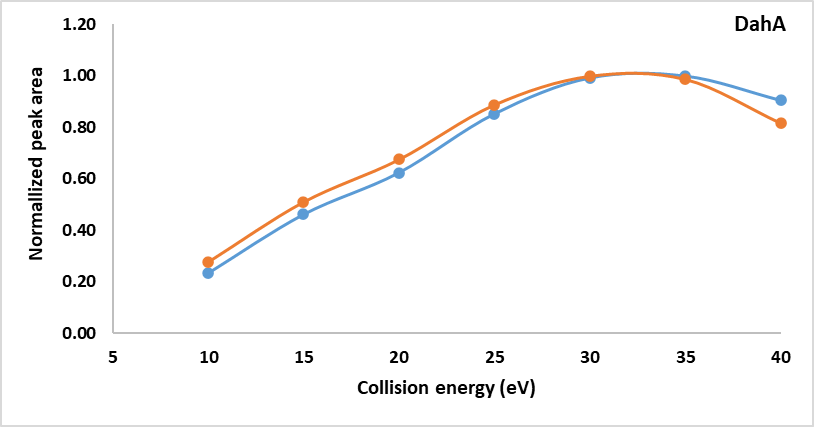 | 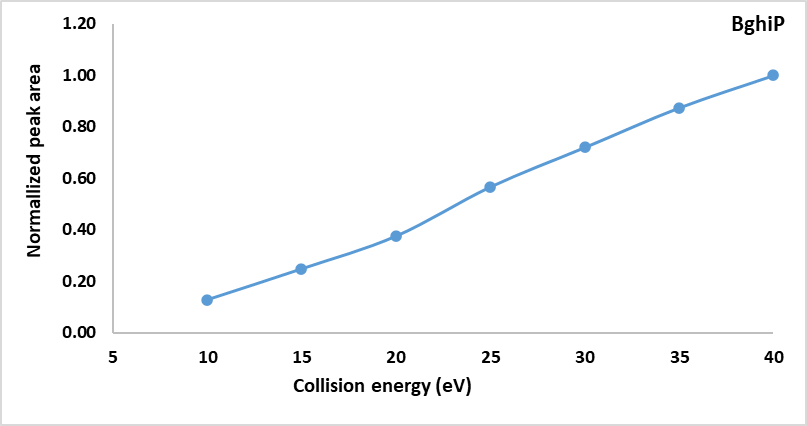 | 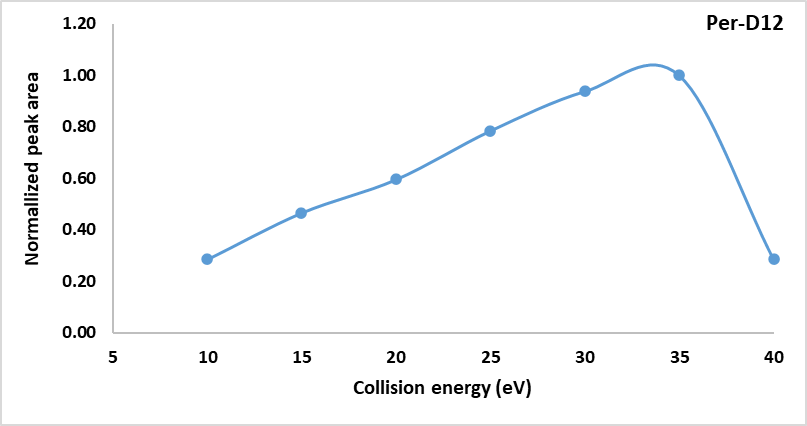 |
| 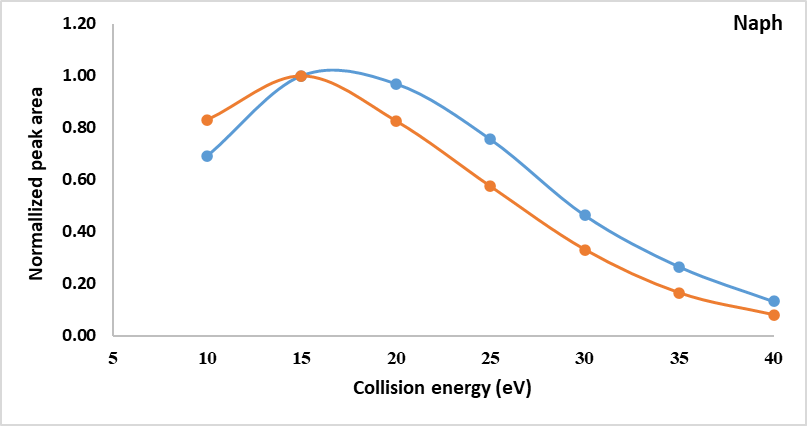 | 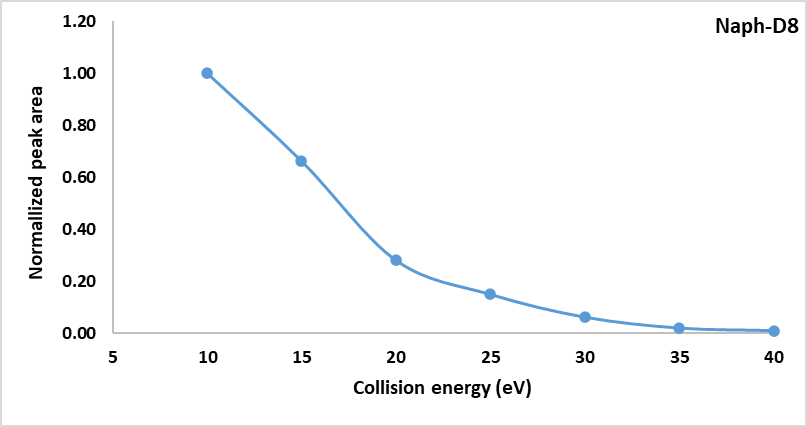 | 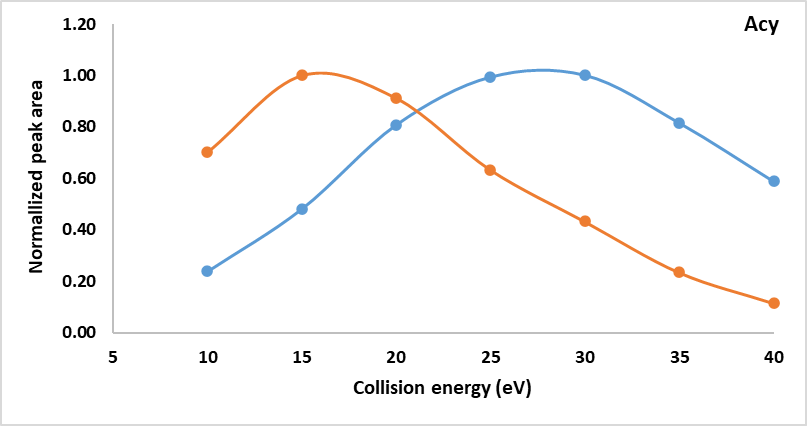 |

Figure S2: Collision energy of 16 PAHs and isotopic labelled internal standards in MS/MS measurement mode
